# Supplementary material for: A committed fourfold increase in ocean oxygen loss
Source: Nat Commun. 2021 Apr 16;12:2307. doi: 10.1038/s41467-021-22584-4 (PMC8052459; doi:10.1038/s41467-021-22584-4)
Supplement: Supplementary file 1 — Supplementary Information [file 41467_2021_22584_MOESM1_ESM.pdf]

Supplementary Information

A committed fourfold increase in ocean oxygen loss

Andreas Oschlies[1,2*]

Affiliations:

[1]GEOMAR Helmholtz Centre for Ocean Research Kiel, Düsternbrooker Weg 20, 24105 Kiel, Germany.

[2]Kiel University, 24098 Kiel, Germany.

*e-mail: aoschlies@geomar.de.

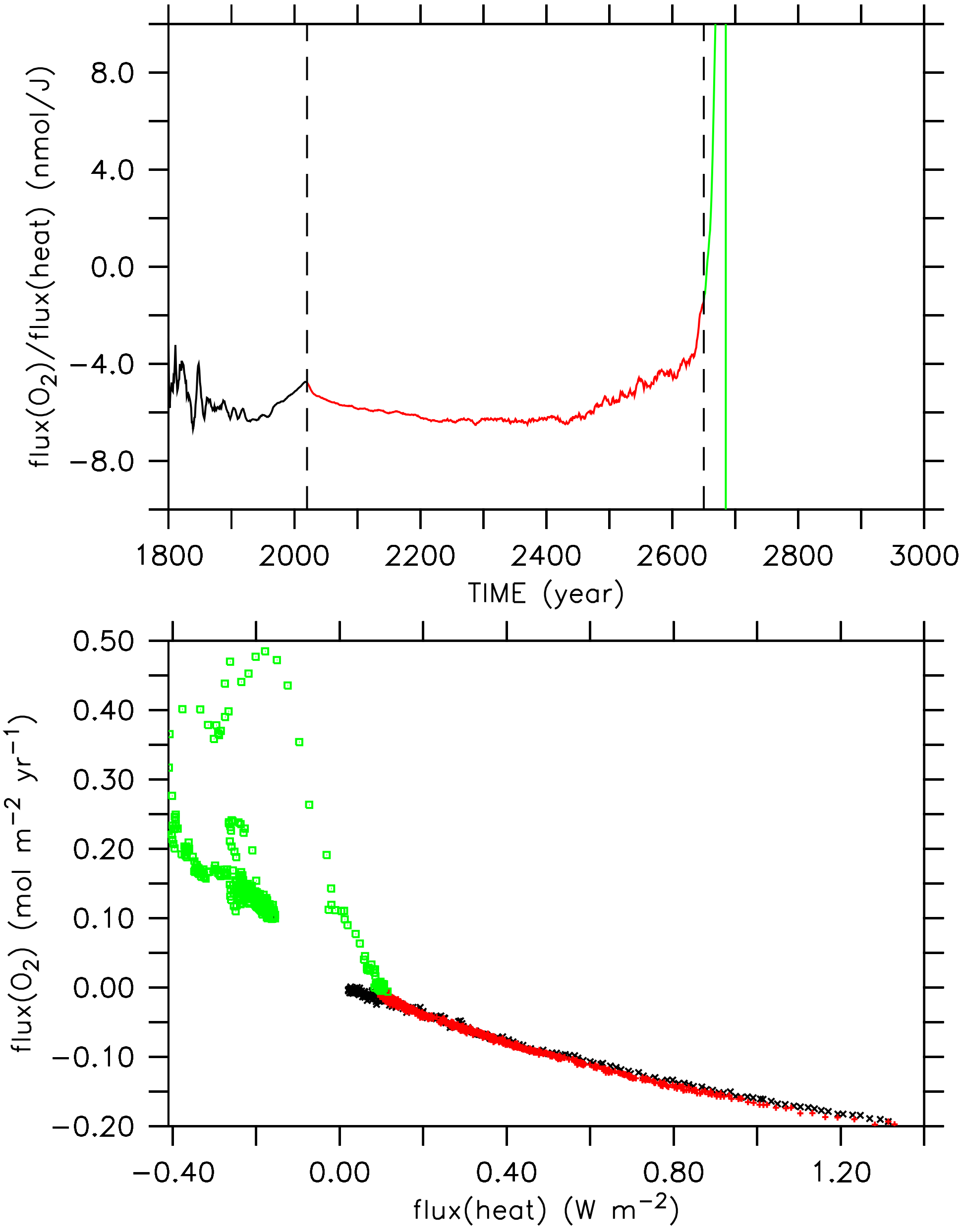

Supplementary Figure 1: Top: Ratio of simulated annual-mean global air-sea fluxes of oxygen and heat, with positive fluxes directed into the ocean. Color code refers to the periods 1800-2020 (black), 2021-2650 (red), and 2651-3000 (green). Bottom: Annual mean air-sea oxygen fluxes versus heat fluxes.

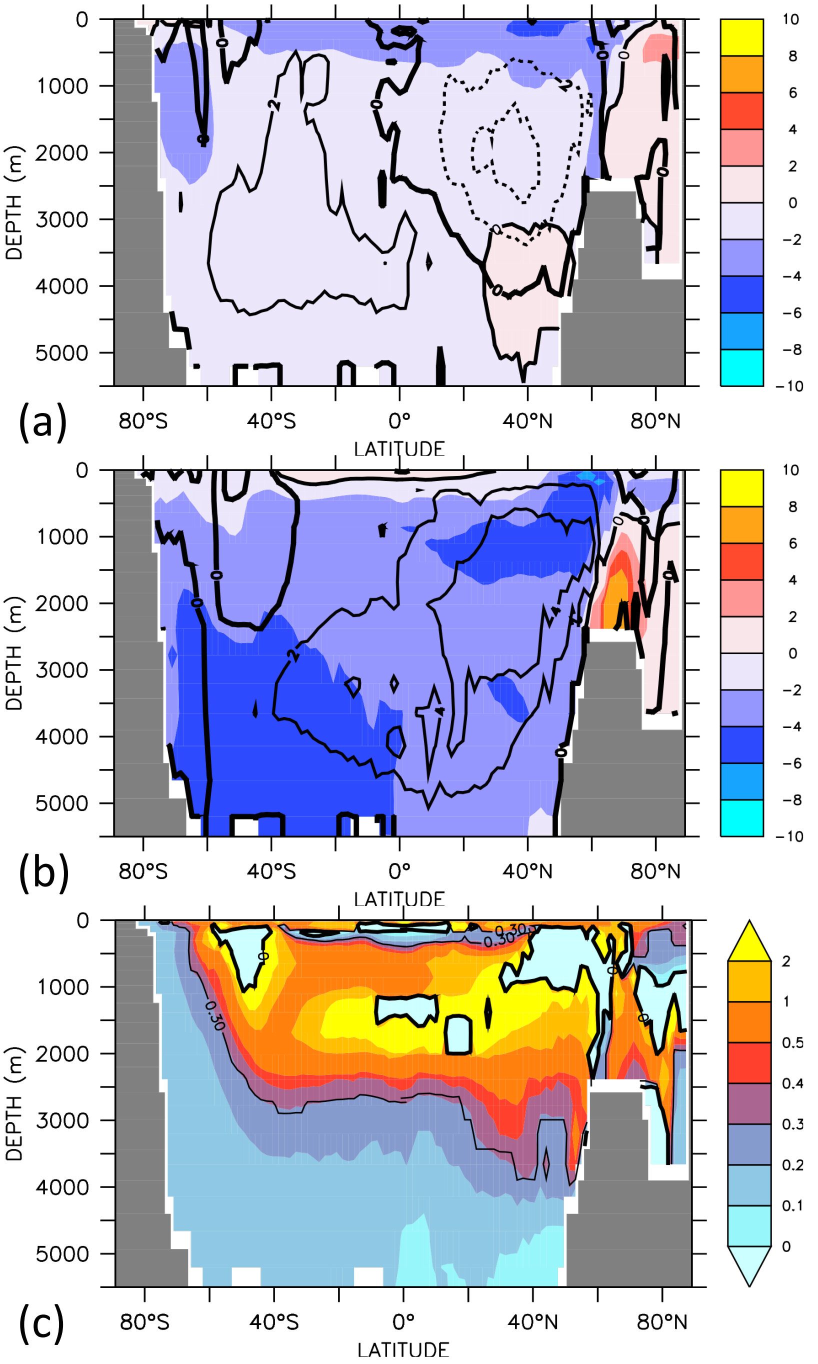

Supplementary Figure 2. Zonally averaged change in the abiotic oxygen tracer (a) year 2020 minus year 1800, (b) year 2650 minus year 2020. Units are mmol/m^3. Contours are changes in the overturning stream function (units Sv, with increments of 2 Sv) over the same time intervals. (c) Ratio of zonally averaged change in abiotic oxygen to zonally averaged change in oxygen (year 2650 minus year 2020). The 0.3 isoline indicates the global mean ratio.

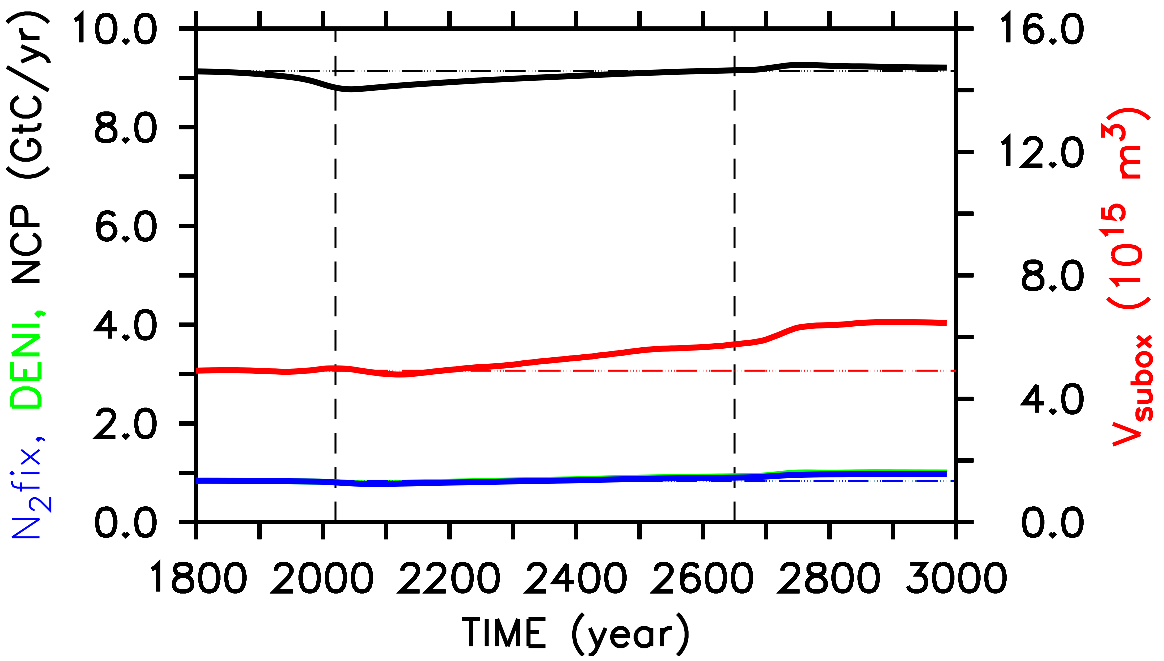

Supplementary Figure 3: Temporal evolution of globally integrated Net Community Production (NCP, black), denitrification (green), almost identical to nitrogen fixation (blue), and the volume of waters with oxygen concentrations lower than 5 mmol m^{-3} (red).

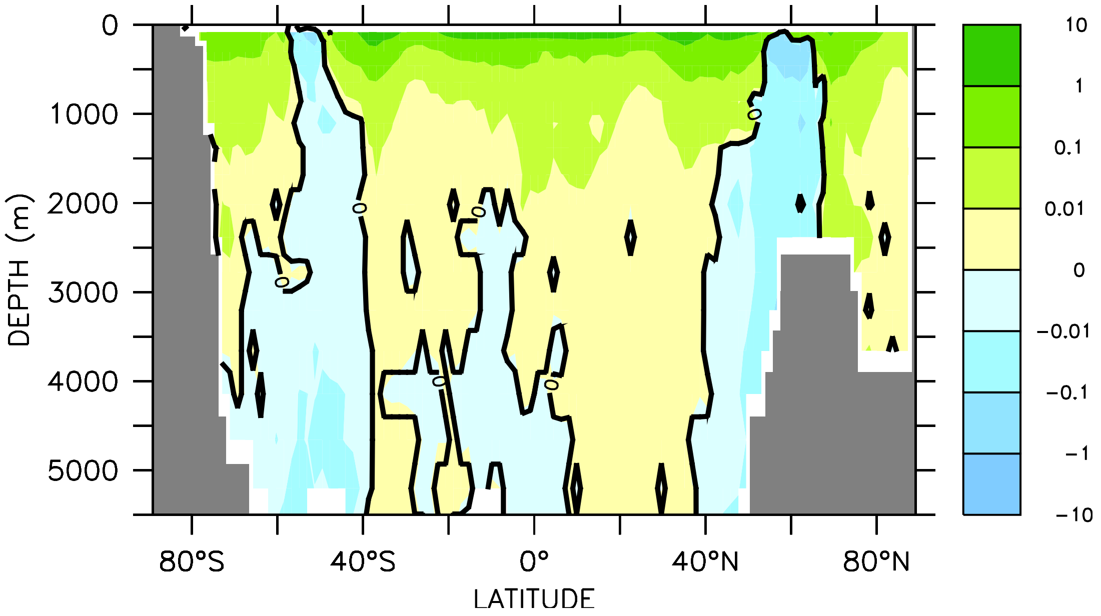

Supplementary Figure 4: Zonally integrated difference in respiratory oxygen consumption, year 2650 minus year 2020. Units are mmol O_2 m^{-3} yr^{-1}.

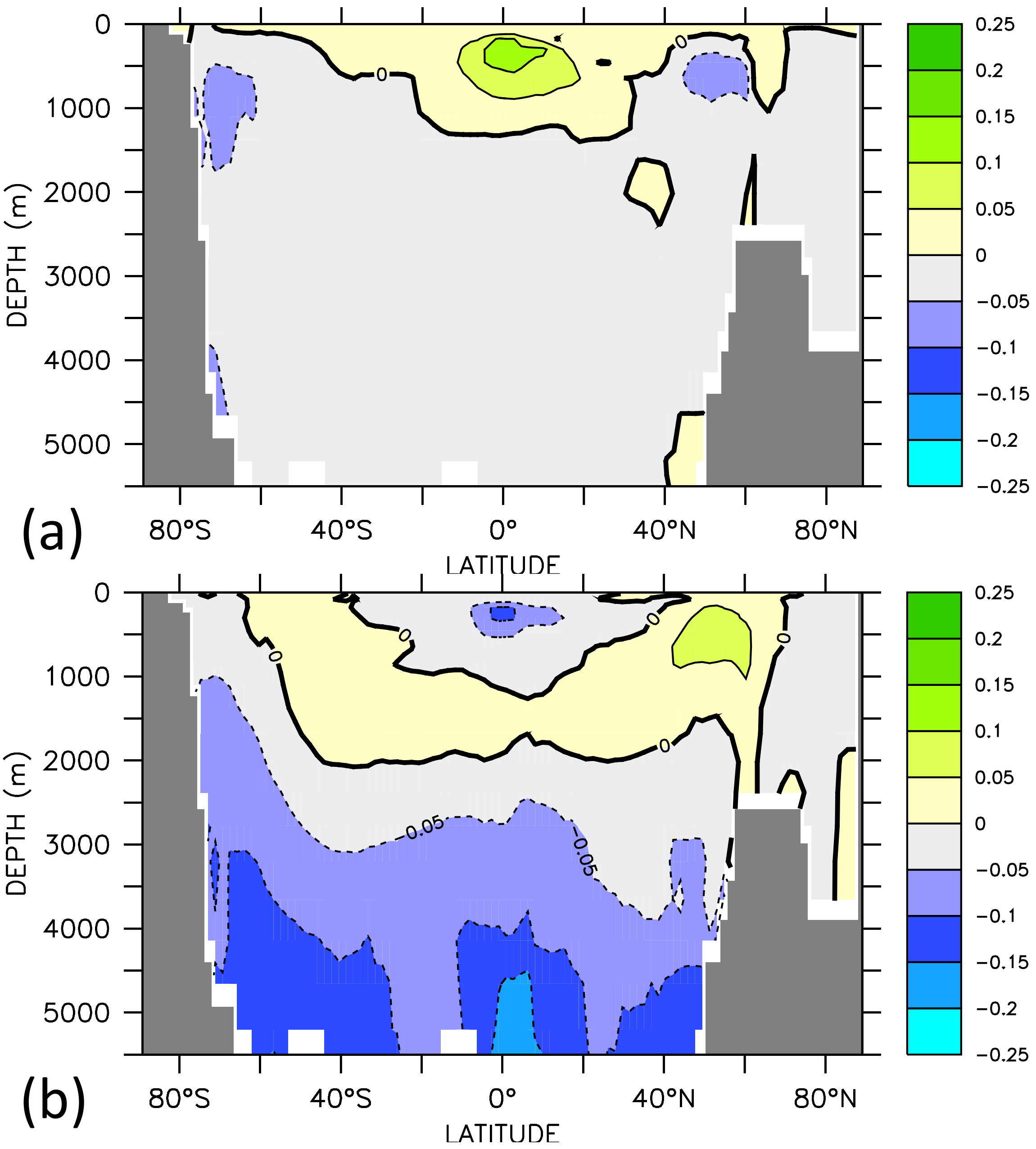

Supplementary Figure 5: Zonally averaged relative changes in the metabolic index Φ (as Figure 5 but for E_0 = -0.2 eV instead of E_0 = 0.4 eV in Figure 5), for (a) year 2020 with respect to year 1800, (b) year 2650 with respect to year 2020.
